# Leonurine ameliorates D-galactose-induced aging in mice through activation of the Nrf2 signalling pathway

**DOI:** 10.18632/aging.101733

**Published:** 2019-09-17

**Authors:** Peng Chen, Fuchao Chen, Ben-hong Zhou

**Affiliations:** 1 Department of Pharmacy, Renmin Hospital of Wuhan University, Wuhan, Hubei 430060, P.R. China; 2 Department of Pharmacy, Dongfeng Hospital, Hubei University of Medicine, Shiyan, Hubei 442008, P.R. China; 3 School of Pharmaceutical Sciences, Wuhan University, Wuhan, Hubei 430071, P.R. China

**Keywords:** aging, D-galactose, leonurine, oxidative stress, Nrf2 pathway

## Abstract

Aging is a complex physiological phenomenon associated with oxidative stress damage. The objective of this study was to investigate the potential effects of leonurine on D-galactose-induced aging in mice and its possible mechanisms. In this study, we first tested the antioxidant activity of leonurine *in vitro*. A subcutaneous injection of D-galactose in mice for 8 weeks was used to establish the aging model to evaluate the protective effects of leonurine. The results showed that treatment with 150 mg·kg^-1^ leonurine could improve the mental condition, organic index, and behavioural impairment; significantly increase the activities of antioxidative enzymes including SOD, CAT, and T-AOC; and ameliorate the advanced glycation end product (AGE) level and histopathological injury. Furthermore, the Western blotting data revealed that leonurine supplementation noticeably modulated the suppression of the Nrf2 pathway and upregulated the downstream expression of HO-1 and NOQ1 in aging mice. Additionally, leonurine treatment activated Nrf2 nuclear translocation in both aging mice and normal young mice, and the expression levels of Nrf2 in normal young mice was higher than those in naturally aging mice. In conclusion, our findings suggest that leonurine is a promising agent for attenuating the aging process, and the underlying molecular mechanisms depend on activating the Nrf2 pathway.

## Introduction

Aging is a biological process influenced by a variety of complex factors, such as heredity, environmental conditions and lifestyle [1]. As aging intensifies, various physiological functions of the body also began to decline, which is characterized by depression, forgetfulness, shortness of breath, and swelling [2]. These symptoms are extremely detrimental to the health and quality of life of the human body. The free radical theory of aging says that oxidative damage caused by reactive oxygen species (ROS) or free radicals induced from cell metabolism is a key factor that accelerates aging [3]. Although the mechanisms of aging are complex and mysterious, plenty of recent research has indicated that oxidative stress is involved in the aging process [4]. Previous studies have demonstrated that the antioxidant defence system regulates the intracellular redox balance in the cell and ameliorates the status of inflammation, senescence and ROS [5]. Therefore, stress defence plays an important role during the aging process, and resistance to oxidative stress maybe a promising therapeutic approach to delay or treat age-associated diseases [6].

Nuclear-erythroid 2-related factor 2 (Nrf2) is a key transcription factor that regulates multiple antioxidants [7]. Upon exposure of human cells to oxidative stress, Nrf2 translocates into the nucleus to bind to antioxidant response elements (AREs), which activates phase II antioxidative enzymes such as haem oxygenase-1(HQ-1) and NADPH quinone oxidoreductase 1(NQO1) [8,9]. Accumulating evidence has demonstrated that Nrf2 knock-out mice display an extreme susceptibility to oxidative damage due to a severe lack of coordinated gene regulatory procedures, which suggested that Nrf2 plays an important role in the antioxidant defence system [10]. It has also been reported that Nrf2 expression and activity decreased in aging mammals, and Nrf2 activation was proposed as a potential therapeutic target in aging [11,12].

**Figure 1:**
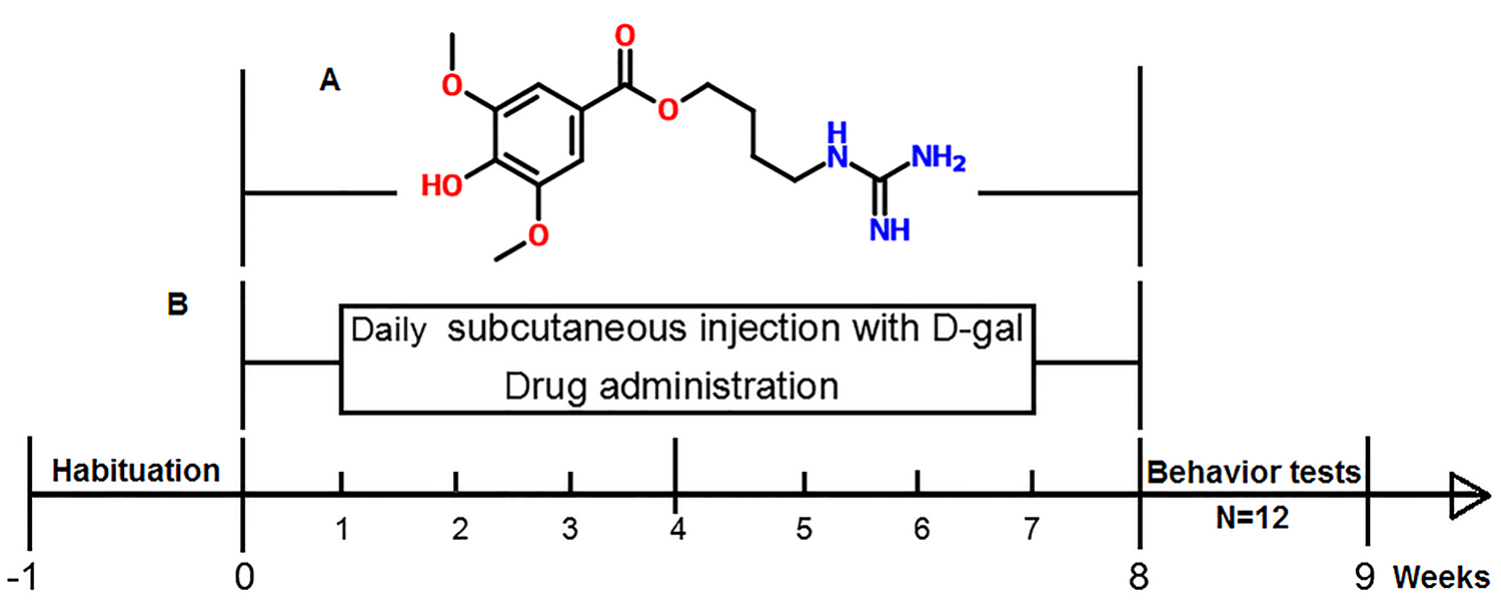
(**A**) Molecular structure of leonurine. (**B**) Diagram illustrating the process of the research.

Leonurine (C_14_H_21_N_3_O_5,_ 4-hydroxy-3, 5-dimethoxy, 4-[aminoiminomethyl) amino] butyl ester, the chemical structure shown in Fig. 1A), also known as benzoic acid, is an active alkaloid from food and a traditional Chinese medicine named Herba leonuri [13,14]. Modern pharmacological studies have reported that leonurine has a wide range of biological and pharmacological activities such as antioxidative, anti-inflammatory, antiapoptotic, anti-diabetic, and improving the circulatory system effects [15,16]. It has recently been demonstrated that leonurine has a neuroprotective effect on ischaemia/reperfusion-induced mitochondrial dysfunction in the rat cortex, which are ascribed to its antioxidant functions [17]. However, there are still no scientific reports to date focused on the protective effects of leonurine on D-gal-induced aging in mice and its anti-aging molecular mechanisms.

Thus, the present study aimed to estimate the anti-aging effects of leonurine in D-gal-induced aging mice and explore the underlying mechanisms in the aging process [18]. We hypothesized that leonurine could ameliorate the oxidative damage and that the Nrf2 signalling pathway is involved in its anti-aging effects to delay the aging process.

## RESULTS

### The antioxidant activity of leonurine *in vitro*

**Figure 2:**
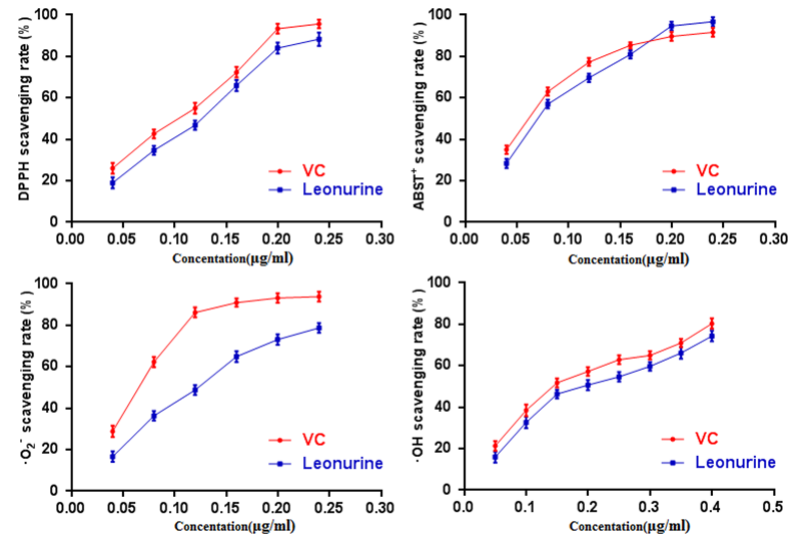
**Effect of leonurine on antioxidant activity *in vitro*.** (**A**) DPPH scavenging assay. (**B**) ABTS^+^ scavenging assay. (**C**) O^2^· scavenging assay. (**D**)·OH scavenging assay. The data are expressed as means ± SD (*n* = 5).

Oxidative stress (OS), which is a negative effect produced by reactive oxygen species (ROS), has been regarded as one of the most significant factors in the ontogeny of many multifactorial diseases, including aging, cancer, Parkinson's disease and diabetes. DPPH, ABTS^+^, O^2^· and ·OH are four kinds of free radicals that are usually used to assess the antioxidant capacity of natural crude drugs and health foods [19,20]. Hence, DPPH, ABTS^+^, O^2^·and ·OH assays were evaluated to assess the antioxidant capacity of leonurine in this study. The results showed that leonurine possessed a good ability to scavenge DPPH radicals in a dose-dependent manner, and the 50% effective concentration (EC_50_) (447.78±4.66 μmol·L^-1^) was lower than that of VC (774.54 ± 6.08 μmol·L^-1^) (Fig. 2A). The results from the ABTS^+^ radical scavenging assay (Fig. 2B) also showed that leonurine exerted a strong activity to scavenge ABTS^+^, and it was found that the EC_50_ of leonurine against ABTS^+^ was 408.36±3.60 μmol·L^-1^_,_ whereas that of VC was 659.09±5.51 μmol·L^-1^. The scavenging effects of leonurine on O^2^·and OH were also determined, and the results showed that the EC_50_ of leonurine and VC for O^2^·(·OH) were 450.16±7.33 (681.82±6.36) μmol·L^-1^ and 707.40±4.95 (1238.63±7.16) μmol·L^-1,^ respectively, indicating that the scavenging rate of leonurine towards O^2^·and OH was comparable to VC (Fig. 2C and 2D).

### Rota-rod test

**Figure 3:**
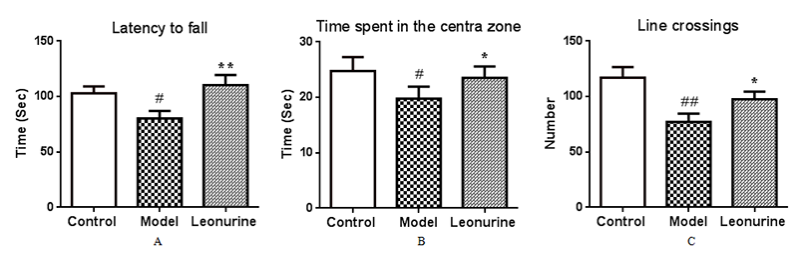
**Effect of leonurine on** (**A**) Rota-rod test and (**B, C**) open ﬁeld test. (**A**) Latency to fall from the Rota-rod. (**B**) The time spent in the central zone. (**C**) The total number of crossing lines in the open area. All data are expressed as means ± SD (*n* = 12). ^#^*P* < 0.05 and ^##^*P* < 0.01 vs control group; *P < 0.05 and **P < 0.01 vs model group.

The accelerating Rota-rod experiments were performed to investigate the effects of leonurine on motor coordination in aging mice induced by D-gal. As observed in Fig. 3A, the model group mice were showed impaired motor coordination functions with a significant decrease in latency to fall from the Rota-rod compared to the control group (*P<*0.01). However, after treatment with leonurine, motor function was significantly promoted when compared to the aging group (*P<*0.01).

### Open-field activity

The effects of leonurine on spontaneous activities in aging mice were evaluated by an open-field study. The results showed that the number of squares crossed and the time spent in the central square in the model group mice were both lower than the control group (*P* < 0.05, *P* < 0.01), indicating that there was a significant difference in the spontaneous activities between the control and D-gal-treated mice (*P*<0.05 or *P*<0.01) (Fig. 3B and 3C). In contrast, leonurine treatment noticeably ameliorated the spontaneous activity defects (all *P* < 0.05).

### Morris water maze

**Figure 4:**
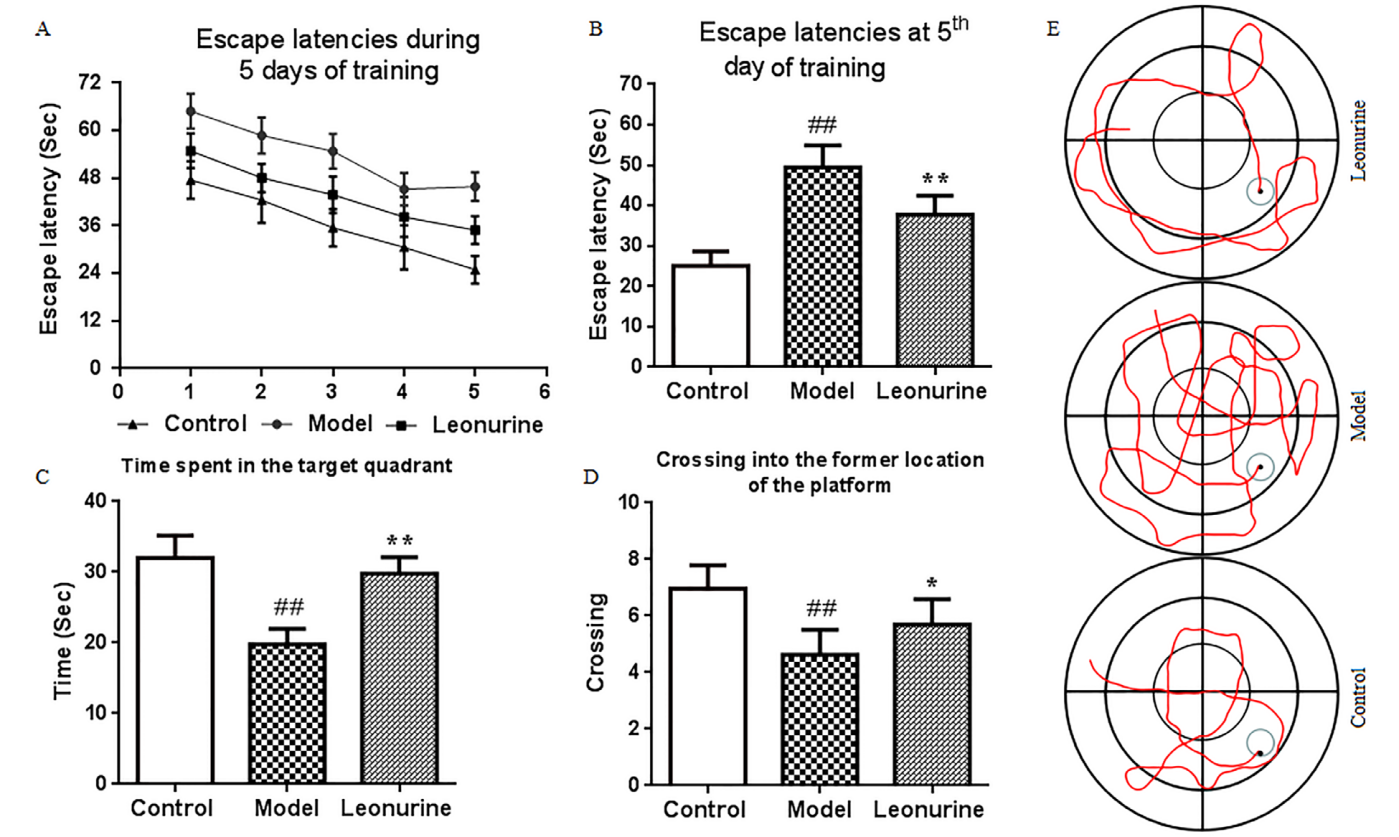
**Effect of leonurine on spatial learning and memory impairment in the MWM task.** (**A**) Escape latencies of the training trial. (**B**) Escape latencies at the 5th day of the training trial. (**C**) Swimming time in the target quadrant during probe test. (**D**) The number of crossings into the former location of the platform during the probe test. (**E**) Swimming trajectory of each group of mice. All data are expressed as means ± SD (*n* = 12). ^#^*P* < 0.05 and ^##^*P* < 0.01 vs control group; *P < 0.05 and **P < 0.01 vs model group.

The Morris water maze was used to assess the effects of leonurine on spatial learning and memory in the aging model mice in this study. As shown in Fig. 4A and 4B, the escape latencies were significantly prolonged after administration of D-gal during the training period (*P*<0.01), and leonurine could attenuate the increase when compared to the aging model group (*P*<0.01). In the probe trial, the time spent in the target quadrant and crossing into the former location of the platform in the D-gal group mice showed great decreases (both *P*<0.01) compared to the control group. However, the poor situation was significantly restored by leonurine treatment (*P*<0.01 or *P*<0.05) (Fig. 4C and 4D). These findings indicated that leonurine could prevent D-gal-induced learning and memory dysfunction in mice.

### Effects of leonurine on mental state, body weight and organ indices

After the 4-week establishment of the aging model, the mice in the normal group were active, sensitive to sound and light, possessed white and shiny hair and their faeces were granular, while the mice in the model group were gaunt, listless, unresponsive and their hair was lustreless. However, these deteriorated symptoms changed in mice from the leonurine group. There was a significant decrease in the body weight and organ coefficient in the control group compared with the D-gal-treated model group when the experiment ended (*P*<0.05 or *P*<0.01, Table 1). After 8 weeks of treatment with leonurine, the organ coefficients for the liver, kidney, thymus, and spleen and the mouse weight were significantly improved (*P*<0.05 or *P*<0.01).

**Table 1:** The effect of leonurine on mental state, body weight and organ indices.

**Group**	**Body weight(g)**	**Organ index (mg/g)**			
		**Liver index**	**Spleen index**	**Thymus index**	**Kidney index**
**Control**	36.94±1.46	37.12±0.78	1.89±0.21	1.49±0.21	10.72±0.53
**Model**	28.79±1.24^##^	29.06±1.22^##^	1.62±0.23^#^	1.18±0.12^#^	8.14±1.29^#^
**D-gal+leonurine**	36.74±1.31^△△^	36.14±0.75^△△^	1.94±0.31^△^	1.46±0.17^△^	9.91±0.53^△^

Data are presented as mean±SD from each group (n=12, mean±SD). ^#^*P* < 0.05 and ^##^*P* < 0.01 vs control group; ^△^*P* < 0.05 and ^△△^*P* < 0.01 vs model group.

### Histopathology analysis

**Figure 5:**
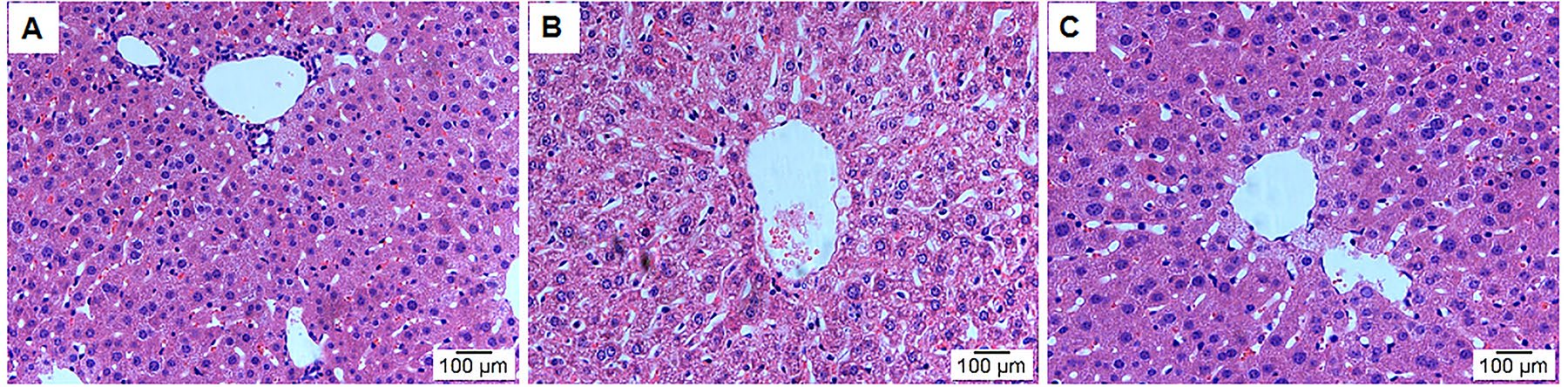
**Effect of leonurine treatment on liver histopathological alterations.** (**A**) Control group; (**B**) Model group; (**C**) Leonurine group. H&E staining, magnification 200×.

HE staining was used to analyse the effects of leonurine on the liver tissue histopathological features from aging mice. As shown in Fig. 5, the model group showed significant liver damage, including hepatocellular hydropic degeneration, necrosis, and inflammatory cell infiltration. However, there was an obvious increase in hepatocellular hydropic degeneration and necrosis upon treatment with leonurine compared to the mice in the model group. These findings suggested that hepatic pathological changes could be ameliorated by leonurine intervention.

### Effects of leonurine on antioxidant parameters *in vivo*

**Figure 6:**
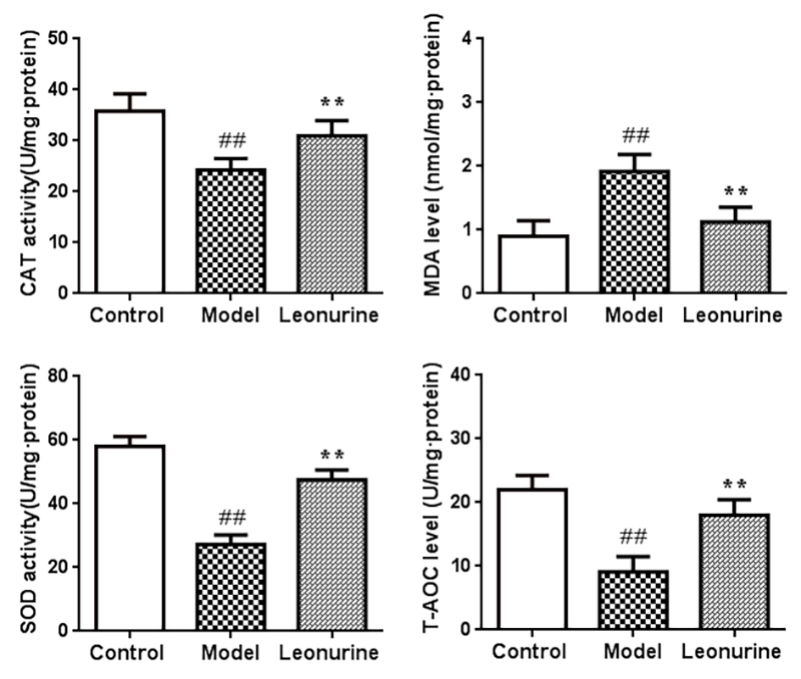
**Effect of leonurine on SOD, MDA, CAT, and T-AOC in mice.** Data are presented as mean±SD from each group (n=12, mean±SD). ^#^*P* < 0.05 and ^##^*P* < 0.01 vs control group; *P < 0.05 and **P < 0.01 vs model group.

It has been reported that oxidative damage is closely related to the aging process. Therefore, we evaluated the effects of leonurine on oxidative stress induced by D-gal and detected the levels of SOD, MDA, CAT, and T-AOC. As shown in Fig. 6B, compared to the control group mice, there was an obvious increase in the levels of MDA in the liver tissue from the model group (*P*<0.01). However, the MDA levels could be significantly inhibited after leonurine supplementation (*P*<0.01). Additionally, the activities of SOD, CAT, and T-AOC in livers from the model mice were significantly lower than those in the control group (*P*<0.01), and leonurine significantly improved these biochemical indices, which was obviously different from the model group (all *P*<0.01) (Fig. 6A, 6C and 6D). Our results also showed that leonurine could attenuate oxidative stress in D-gal-induced aging mice by restoring the antioxidant defence system.

### Measurement of ALT and AST levels

**Figure 7:**
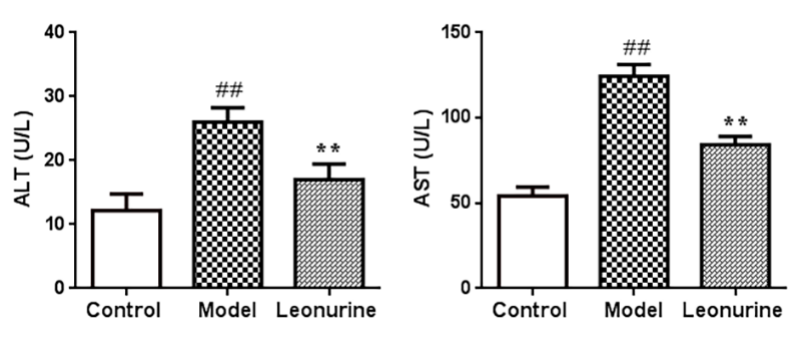
**Effect of leonurine on ALT and AST levels in mice.** Data are presented as mean±SD from each group (n=12, mean±SD). ^#^*P* < 0.05 and ^##^*P* < 0.01 vs control group; *P < 0.05 and **P < 0.01 vs model group.

The ALT and AST levels in serum were estimated to assess the effects of leonurine on hepatic function and liver injury induced by D-gal. As shown in Fig. 7, ALT and AST levels in the serum significantly increased after treatment with D-gal in aging mice when compared with the control group (both *P*<0.01). As a result, after leonurine supplementation, the levels of ALT and AST were significantly restored in the treatment group compared to the model group (both *P*<0.01) (Fig. 7A and 7B). These findings showed that leonurine could improve hepatic function and liver damage.

### AGE level determination

**Figure 8:**
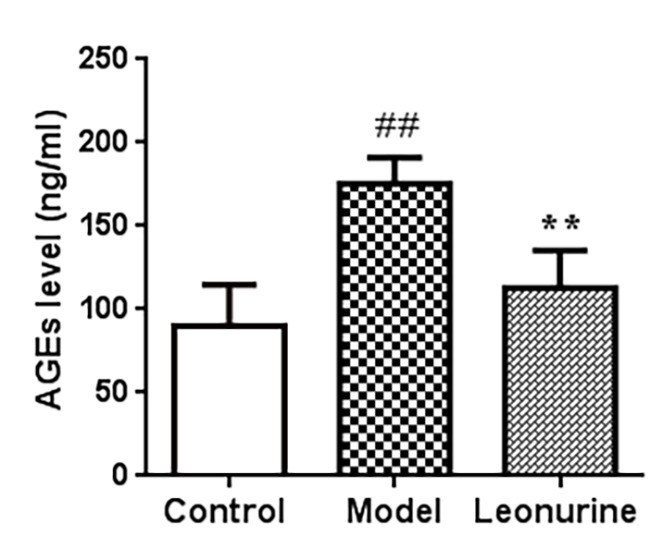
**Effect of leonurine on the AGEs level in serum of mice.** Data are presented as mean±SD from each group(n=12, mean±SD). ^#^*P* < 0.05 and ^##^*P* < 0.01 vs control group; *P < 0.05 and **P < 0.01 vs model group.

The formation of AGEs is a part of normal metabolism and aging, but the presence of excessively high levels of AGEs in tissues and serum could promote oxidative stress; thus, AGEs are widely considered as a pivotal biomarker for senescence. Fig. 8 shows that the levels of AGEs in the serum in the model aging mice increased significantly when compared to the control group (*P*<0.01) and that leonurine significantly increased the AGEs level, which was obviously different from the model group (*P*<0.01).

### Effects of leonurine on Nrf2 expression in aging mice

**Figure 9:**
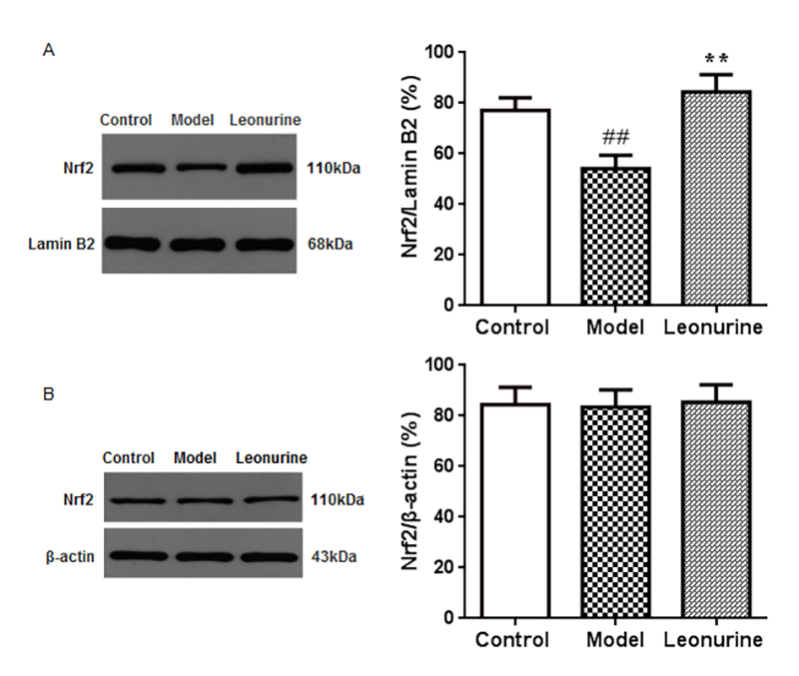
**Effect of leonurine on the expressions of Nrf2 protein** in the nuclear fraction (**A**) and cytosolic fraction (**B**) using western blot analysis. Data are presented as mean ±SD from each group(n=6, mean±SD). ^#^*P* < 0.05 and ^##^*P* < 0.01 vs control group; *P < 0.05 and **P < 0.01 vs model group.

To address whether the Nrf2 pathway was associated with D-gal-induced aging, we investigated the expression of Nrf2 protein in the liver tissue nuclear and cytosolic fractions (Fig. 9A and 9B). The results of our study showed that the expression levels of Nrf2 in the aging model group were lower than in the control group (*P* <0.01) and that the leonurine group significantly attenuated the downregulation of Nrf2 protein expression (*P<*0.01). However, there were no significant effects of leonurine treatment on Nrf2 protein expression in the cytosolic fraction (*P*>0.05). These data suggested that the effects of leonurine on D*-*gal-induced aging mice were associated with the promotion of Nrf2 protein to translocate to the nuclear fraction from the cytosolic fraction.

**Figure 10:**
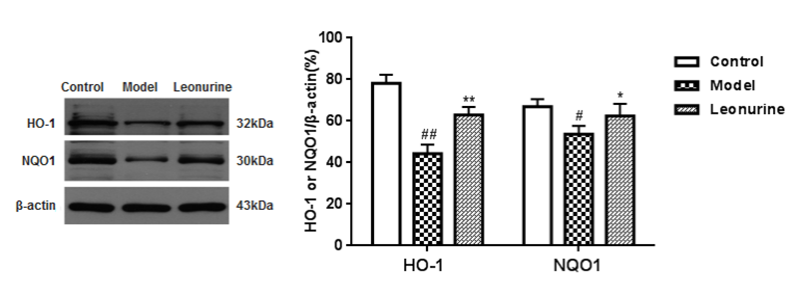
**Effect of leonurine on the expressions of HO-1 and NQO1.** Data are presented as mean±SD from each group (n=6, mean±SD). ^#^*P* < 0.05 and ^##^*P* < 0.01 vs control group; *P < 0.05 and **P < 0.01 vs model group.

**Figure 11:**
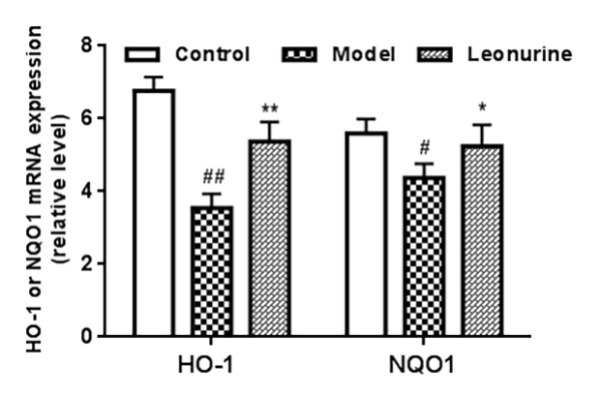
**The expression of HO-1 and NQO1 in different groups was analyzed through quantitative RT-PCR.** Data are presented as mean±SD from each group (n=6, mean±SD). ^#^*P* < 0.05 and ^##^*P* < 0.01 vs control group; *P < 0.05 and **P < 0.01 vs model group.

To confirm that whether the beneficial effects of leonurine are involved in Nrf2 pathway activation, we detected the expression of the phase II antioxidant enzymes HO-1 and NQO1. As shown in Fig. 10, leonurine administration significantly enhanced HO-1 and NQO1 expression. Furthermore, HO-1 and NQO1 mRNA levels were also detected. As described in Fig. 11, HO-1 and NQO1 mRNA levels were significantly decreased in the D-gal-induced aging model when compared with the normal control group (*P* < 0.05 or 0.01). In contrast, leonurine treatment significantly increased HO-1 and NQO1 mRNA levels (*P* < 0.05 or 0.01). These findings have demonstrated the role of leonurine in activating the Nrf2 pathway.

### Effects of leonurine on the expression of Nrf2 in normal mice

**Figure 12:**
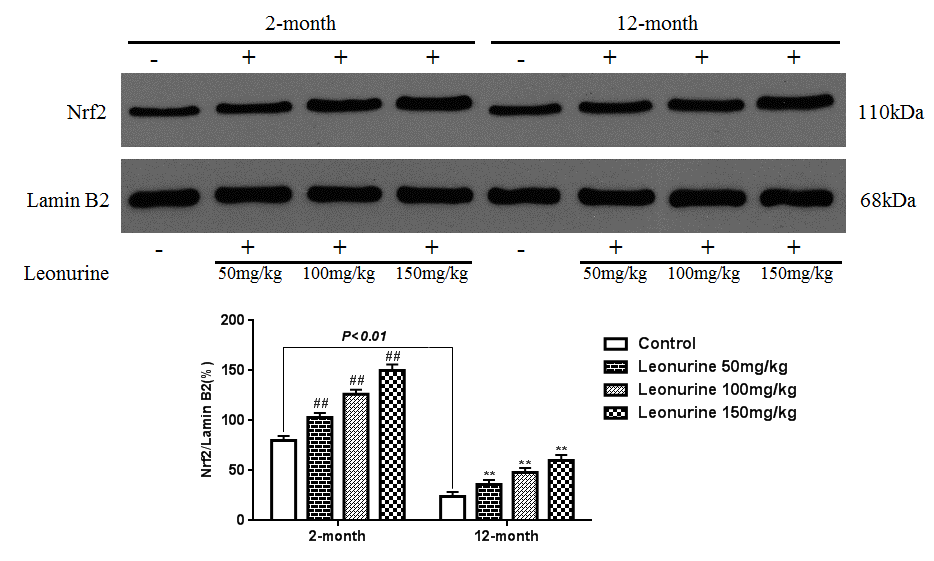
**Effect of leonurine on the expression of the Nrf2 protein from normal young mice (2-month) and natural senile mice (12-month) after leonurine administration for 2 months in nuclear.** Data are presented as mean±SD from each group (n=6, mean±SD). ^#^*P* < 0.05 and ^##^*P* < 0.01 vs control group; *P < 0.05 and **P < 0.01 vs model group.

**Figure 13:**
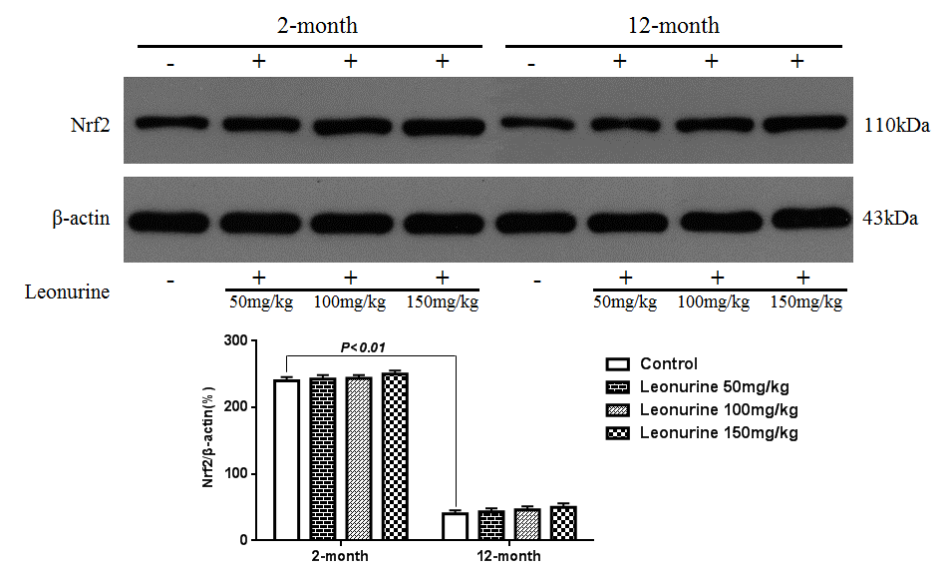
**Effect of leonurine on the expression of the Nrf2 protein from normal young mice (2-month) and natural senile mice (12-month) after leonurine administration for 2 months in cytosolic fraction.** Data are presented as mean±SD from each group (n=6, mean±SD). ^#^*P* < 0.05 and ^##^*P* < 0.01 vs control group; *P < 0.05 and **P < 0.01 vs model group.

To further study the effects of leonurine on normal young mice and natural aging mice, 2-month- and 12-month-old mice were treated with leonurine, and Nrf2 nuclear translocation was measured in these mice. As shown in Fig. 12, leonurine significantly increased the levels of Nrf2 nuclear translocation in both the 2-month- and 12-month-old mice in a dose-dependent manner (*P*<0.01 and *P*<0.01), whereas the difference between the leonurine and control mouse groups in the expression of Nrf2 in the cytosolic fractions was not significant (*P*>0.05, Fig. 13). Furthermore, we found that the 12-month-old mice experienced a significant decrease in Nrf2 expression levels in both the nuclear and cytosolic fractions compared to the 2-month-old mice (*P*<0.01 and *P*<0.01). These data confirmed the hypothesis that Nrf2 expression levels in young rodents were higher than in their aging partners [21].

## DISCUSSION

Aging is a multifactorial process that has gained extensive attention and interest [22]. Accumulating evidence has shown that aging is an important risk factor for degenerative diseases, such as cancers, cardiovascular and cerebrovascular diseases, diabetes, liver disease and dementia, and especially Alzheimer’s disease (AD), which is one of the main diseases that harms the health of the older generation [23,24]. Most elderly people show severe a decline in physiological function, such as difficulties in learning and memory, decreases in cognitive functions, and behaviour disorders, but the underlying molecular mechanisms are not fully understood [25]. Numerous studies have shown that oxidative stress plays a key role in the aging process [26]. The imbalance between the generation and elimination of reactive oxygen species (ROS) can lead to the impairment of various biomolecular processes, resulting in the acceleration of aging and the occurrence and development of AD [27].

D-Galactose (D-gal) is a reducing sugar normally present in the body that can be converted into aldose and hydroperoxide by galactose oxidase at high concentrations, resulting in the generation of oxygen-derived free radicals, which can also react with the amino groups in proteins and peptides to form AGEs [28,29]. Large amounts of evidence have confirmed that chronic systemic administration of D-gal can induce aging-related changes, including the corruption of spatial learning, memory impairment, lowered antioxidant enzyme activities and increased production of ROS [30]. Therefore, chronic injection of D-gal has been demonstrated as an effective way to establish an animal model for studying cognitive disorders and aging, as well as drug testing [31]. The present study evaluated the effects of leonurine on D-gal-induced aging mice, and the results demonstrated that leonurine prevented aging. To our knowledge, this study provided evidence for the first time that (1) leonurine administration could improve behavioural performance in aging mice and (2) leonurine treatment could protect mice against D-gal-induced oxidative damage in an aging model. Moreover, the protective effects of leonurine on aging mice are closely correlated to activation of the Nrf2 signalling pathway [32].

The present study investigated the effects of leonurine on D-gal-induced aging mice for the first time. First, we observed that leonurine possessed a good antioxidant activity in DPPH, ABTS^+^, O^2^· and ·OH assays. We also found that leonurine treatment could substantially ameliorate spatial location, learning and memory impairments. The mice were sacrificed after the behaviour experiments to assay for biochemical indicators and histological assessment, and the results showed that leonurine treatment could attenuate pathological damages to the liver and remarkably reduce CAT, MDA, SOD, and T-AOC levels as well as AGE products in aging mice. These findings suggested that leonurine has potential anti-aging effects. The present study also demonstrated that the Nrf2 pathway was one of the mechanisms involved in the effects of leonurine against the aging induced by D-gal. Further investigations showed that the Nrf2 pathway was potently activated by increasing Nrf2 transcription levels, which facilitated the transcription of downstream target genes such as HO-1 and NQO1 in the aging model mice [33].

It has been demonstrated that excessive complementation with D-gal may accelerate the aging process by increasing the generation of ROS metabolized from D-gal, including mitochondrial damage and the accumulation of AGEs, which are harmful post-translational protein modiﬁcations that trigger excess oxidative stress [34,35]. Recently, accumulating evidence has suggested that the interaction between AGEs and its cell surface receptors, such a receptor (biochemistry) nicknamed RAGE, implicates AGEs in the progression of age-related diseases including neurodegenerative disorders; mediates the activation of the nuclear factor kappa B (NF-κB), which controls several genes involved in immunity, inflammation, cell apoptosis, and astrocytes activation; and ultimately results in atherosclerosis, diabetes mellitus and neuropathy [36,37]. The present study demonstrated that leonurine supplementation markedly downregulated the levels of AGEs in serum from aging mice, suggesting that leonurine effectively prevented the aging induced by D-gal.

It was widely accepted that the redox equilibrium is involved in the aging process. In antioxidant defence systems, SOD and CAT are a group of antioxidative enzymes that are commonly used to assess the severity of oxidative stress *in vivo* [38]. SOD is the first gatekeeper in the antioxidant defence system and can catalyse the reduction of O^.-^ to H_2_O_2,_ the latter of which is metabolized into water and oxygen by CAT catalysis. T-AOC represents the vitality of all the antioxidant enzymes and MDA is one of the most important products in membrane lipid peroxidation, which can aggravate organ damage and has been commonly used as an indicator of aging [39–41]. In the present work, the results showed decreased SOD, CAT, and T-AOC levels as well as increased MDA in D-gal-induced aging mice. However, leonurine treatment showed beneficial effects in improving the activities of antioxidant enzymes and MAD contents in mouse livers. These data suggest that leonurine can protect mouse liver tissues against D-gal-induced oxidative damage [42]. Moreover, histopathological changes in the liver and the activities of some serum enzymes, which are useful biomarkers of liver injury, were also evaluated in this study. Our results suggest that D-gal injection results in conspicuously elevated ALT and AST levels, hepatocyte apoptosis and inflammatory cell infiltration in hepatic tissue. However, these histopathological changes and increased levels of serum enzymes were ameliorated after long-term intervention with leonurine, signifying that leonurine had protective effects on D-gal-induced hepatic dysfunction *in vivo* during the experimental period.

Leonurine not only showed strong antioxidant activity *in vitro* but also exhibited protective effects against D-gal-induced damage by activating antioxidant defence systems. Nrf2, a critical signalling pathway for oxidative stress, could regulate the expression of several phase II antioxidant enzymes by interacting with antioxidant response elements (AREs) [43,44]. In our study, D-gal administration suppressed Nrf-2 translocated translocation to the nucleus, whereas leonurine treatment demonstrated a protective effect in aging mice by inhibiting this suppression, suggesting that a key anti-aging property of leonurine is to facilitate Nrf2 nuclear translocation in mice [45]. It has been proven that modification of Kelch-like ECH-associated protein 1 (Keap1) cysteines leads directly to upregulation of AREs and Nrf2 activation, which plays a vital role in the regulation of cellular defence [46]. Under normal physiological conditions, Nrf2 is confined to the cytoplasm, binds to Keap1, and is rapidly degraded through the hydroxylation-ubiquitination-proteasome axis [47]. When exposed to electrophiles or oxidative stress, Nrf2 dissociates from KEAP1 and translocates into the nucleus to transactivate AU-rich element (ARE)-bearing genes and phase II detoxifying enzymes including HO-1and NQO1, which suppress oxidative stress [48,12]. Hence, the expression of downstream target genes is consistent with Nrf2 nuclear translocation, which not only confirms the involvement of the Keap1-Nrf2-ARE pathway but also showed the pivotal role of HO-1 and NQO1 in maintaining redox homeostasis [49].

By analysing the chemical structure of leonurine, we found that leonurine contained a phenolic hydroxyl, a thiol-reactive electrophilic centre that we believe could easily oxidize quinone intermediates and may covalently modify Cys residue(s), leading to the dissociation of Nrf2 from the Keap1-Nrf2 complex and the subsequent activation of Nrf2 [50,51]. Therefore, it is tempting to speculate that leonurine might activate Nrf2 by inhibiting Keap1 protein to promote Nrf2 dissociation from the Nrf2-Keap1 complex [52]. The results from this study showed that the Nrf2 signalling pathway is activated in both aging and normal young mice and that the expression of downstream antioxidant proteins was also enhanced in aging mice after the oral administration of leonurine. Moreover, leonurine treatment attenuated the oxidative damage induced by D-gal by accelerating Nrf2 nuclear translocation. Additionally, our research also discovered that the expression of Nrf2 in normal young mice was higher than in naturally aging mice, suggesting that the Nrf2 pathway is an important target and promising strategy to delay the senescence process [53].

## CONCLUSION

In conclusion, we demonstrated that leonurine treatment could significantly ameliorate motor, spontaneous locomotion and cognitive function behaviours and protect mice against D-gal-induced oxidative stress damage. Additionally, the beneficial effects of leonurine in aging mice may be responsible for awakening the endogenous antioxidant system by activating the Nrf2 signalling pathway. The above findings demonstrate that leonurine is a potential agent for providing therapeutic strategies to delay the aging process.

## MATERIALS AND METHODS

### Reagents and drugs

D-galactose was purchased from Beijing Guangyuan Hengxin Technology Development Co. Ltd. (Beijing, Cat: T8150, purity > 99%) and the leonurine standard was obtained from Shanghai Aladdin Biochemical Technology Co. Ltd. (Shanghai, China, Cat: 149-91-6, purity > 98%). The commercial kits used for the determination of total superoxide dismutase (T-SOD, Cat: A042-2), malondialdehyde (MDA, Cat: A002-5), catalase (CAT, Cat: A021-4), total antioxidant capacity (T-AOC, Cat: A032-6), aspartate aminotransferase (AST, Cat: A037-4), and alanine aminotransferase (ALT, Cat: A026-3) were supplied by the Nanjing Jiancheng Bioengineering Institute (Nanjing, China). The Advanced glycation end products (AGEs) ELISA kit (Cat: ab11621) was purchased from Shanghai You Ning Wei Biotechnology Co. Ltd. (Shanghai, China). the nuclear-erythroid 2-related factor 2 (Nrf2, Cat: D4020-11), hemeoxygenase-1 (HO-1, Cat: D402012) and Quinone 1 (NQO1, Cat: D402015) antibodies were supplied by Hangzhou Sanofi-Aventis Minsheng Pharmaceutical Co. Ltd. (Shanghai China). All the other chemicals used were of analytical grade and purchased from Guoyao Chemical Reagent Co., Ltd. (Shanghai, China) if not otherwise stated.

### Determination of leonurine antioxidant activity *in vitro*

#### DPPH radical scavenging assay

The assessment of the DPPH radical scavenging capacity of leonurine was performed according to a previous method with minor modifications [54]. Leonurine was dissolved in 50% ethanol and diluted into a series of solutions with 30-200 μg/ml samples. Then, 2 ml of sample solution was mixed with 2 ml of 0.24 mM DPPH solution in 95% ethanol, and the mixtures were fully shaken and placed in the dark for 30 min at room temperature. After that, the absorbance (Abs) of the mixtures was measured at 519 nm via UV visible spectrophotometry. Vitamin C (VC) was used as a positive control in this study. The DPPH radical scavenging activity (%) was defined as follows: scavenging rate (%) = (A_Contro_l-A_Sample_) / A_Control_ ×100%.

#### ABTS^+^ radical scavenging assay

The ABTS^+^ radical scavenging activities of leonurine were determined based on a published report [55]. Briefly, 7.4 mM isometric ABTS^+^ aqueous solution and 2.6 mM K_2_S_2_O_8_ aqueous solution were mixed and held overnight to prepare the ABTS^+^ stock solution. The ABTS^+^ stock solution was diluted 1:40 (v/v) using 95% ethanol to obtain an absorbance of 0.7 ± 0.02 at a wavelength of 734 nm. Then, 1.2 ml ABTS^+^ diluent was mixed with 0.3 ml of leonurine solution at concentrations ranging from 30 to 200 μg/ml and incubated at room temperature for 5 min, after which the absorbance was detected at 734 nm. The scavenging rate (%) was determined with the following formula: [(A_Contro_l-A_Sample_) / A_Control_] × 100%. VC was used as a positive control in this study. Each sample was measured five times.

#### Superoxide anion radical scavenging

The superoxide anion radical assay was performed according to a previously modified method [56]. Briefly, 1 ml of the 30-200 μg/ml leonurine solutions were first diluted to 3 ml with 0.05 mM Tris-HCl buffer (pH 8.2), and then 100 μl pyrogallol was added and mixed well. After 2 minutes, the absorbance of the mixed samples was measured every 30 s for 5 min at 325 nm using a spectrophotometer. The ability to scavenge ·O_2_^-^ radicals was calculated as follows: [(△A_Control_/T - △A_Sample_/T)/ (△A_Control_/T) × 100%, T=5 min].

#### Hydroxyl radical scavenging

The chemicals were mixed in the following order: 75 μl of samples, 150 μl 10 mM thyminose, 150 μl 10 mM EDTA, 150 μl 10 mM FeSO_4,_ 150 μl 10 mM H_2_O_2_, and 525 μl distilled water [57]. The mixture was stirred for one minute, maintained at 37°C for 60 minutes, and then mixed with trichloroacetic acid (TCA) and thiobarbituric acid (TBA) successively. After incubation at 100°C for a 15 minute, the mixture was measured at 520 nm, and the hydroxyl radical scavenging was measured using the following formula: [(A_Control_-A_Sample_) / A_Control]_ × 100%.

### Animals and drug administration

Forty*-*eight male healthy SPF-grade Institute of Cancer Research (ICR) mice (2 months old, weighting 18 to 20 g) were provided by the Wuhan Institute of Biological Products Co. Ltd. (Wuhan China). The mice were housed under constant temperature (21±2°C) and humidity (60%) and exposed to a 12-h dark-light cycle, with free access to water and food. All experimental procedures were approved by the Institutional Animal Care and Use Committee of the Wuhan University (IACUCWU protocol number: SCXK (E) 2013-0004).

After one week of adaptive feeding, the animals were randomly divided into three groups, with 12 mice per group, including the blank control group, aging model group (D-gal), and leonurine-treated group (150 mg·kg^-1^). The mice in the control group were administered normal saline, and the remaining mice were injected subcutaneously with D-gal (150 mg·kg·d^-1^ for 8 weeks), which was dissolved in 0.9% normal saline to establish the aging model. In the meantime, the mice in the leonurine group were administered with leonurine (150 mg per kg body weight, dissolved in 8% Tween 80) *via* gavage for 8 consecutive weeks, and the control group and model animals were subjected to equal volumes of 8% Tween 80 in the same way [58–60]. All mice were fed a standard pellet diet and water was freely available before and after injection. The leonurine dose used in the present study was according to preliminary experiments in our lab. The animal experiments were performed in accordance with the ARRIVE (Animal Research: Reporting *In Vivo* Experiments) guidelines [61].

Additionally, 2-month (n=18) and 12-month (n=18) old male mice were used to investigating the mechanism of leonurine. After oral administration with leonurine (high-dose group, 150 mg/kg, n=6; medium-dose group, 100 mg/kg, n=6; and low-dose group, 50 mg/kg, n=6) for two months, the animals were sacrificed and Western blotting analysis was performed to measure the expression of Nrf2 protein in liver tissues.

### Open field test

The neuropsychiatric changes in the experimental animals were evaluated using the open field test (OFT). The OFT was performed according to a previously described method with slight modifications [62]. The mice were placed in a square arena, and behaviours including the time spent in the centre zone and the total number of squares crossing the OFT were calculated during 5 min trials. After each test, 70% ethanol was used to clean the apparatus and remove any animal odour.

### Rota-rod test

The Rota-rod test was used to assess the effects of leonurine on motor coordination or fatigue resistance in mice and performed according to a modiﬁed previously described method [63,64]. Before the Rota-rod test was performed, all mice were received a training trial. During the training period, the animals were trained to coordinate themselves on the Rota-rod at a constant speed of 25 rpm·min^-1^, and each trial lasted for 2 minutes. If the animals dropped from the Rota-rod, the mouse was immediately placed back on the Rota-rod for training to adapt to the process. Then, the mice underwent Rota-rod testing that consisted of five trials per day for two consecutive days; the incubation period for animals falling off the roller was recorded.

### Morris water maze test

To investigate spatial location learning and memory in all the groups of mice, the Morris water maze test (MWM) was performed according to a described previously method with some modifications [65–67]. The MWM apparatus consisted of a circular water tank (120 cm in diameter and 50 cm in height) that was divided into four quadrants and filled with water (22±1°C) to a depth of 23 cm. A small platform (10 cm in diameter and 22 cm in height) was placed the at the centre of the third quadrant and submerged at a depth of 1 cm below the water surface. The MWM procedure consisted of a place for the navigation training trial and a probe test. During the learning and memory training period, each mouse received four tests per day for four consecutive days. Once the mouse was placed in the water maze, the animal was allowed to swim for 60 seconds to find the hidden platform, and then stay on platform for 15 seconds. If the mouse failed to find the platform within 60 seconds, the animals were guided to the platform and allowed to remain on the platform for 15 s to memorize its location. The probe test was conducted on the day after the training trial, and the mice were permitted to swim freely for 60 s in the tank after the platform was removed.

### Body weight measurement and organ indices analysis

After drug intervention, the mice were injected with 10% chloral hydrate for anaesthesia, and then the animals were weighed and recorded. Blood samples collected from the mouse inferior vena cava were prepared to measure the biochemical parameters. At the end of the experiments, all the mice were euthanized and the liver, kidney, thymus, and spleen tissues from all groups were excised and weighed. Parts of the liver were cut longitudinally and fixed in 4% poly-formaldehyde for pathological studies via haematoxylin-eosin (HE) staining. The rest of the tissues were homogenized and stored at -80°C until further biochemical determinations. The organ indices (OI) were defined as the ratio of the organ weight (OW) to the body weight (BW) and were calculated with the following formula: OI (OW/BW) = organ weight (mg)/body weight(g).

### Histological examination

Formalin-fixed and paraffin-embedded liver tissues were cut into approximately 4-μm thick section, which were stained with HE. For the electron microscopy analysis, 1 × 1 × 1 mm^3^ fragments of hepatic tissues were fixed overnight with cold 2% glutaraldehyde in 0.1 M sodium cacodylate buffer for 2 h in 4 °C. After dehydration in graded ethanol, the pellets were embedded, aggregated and examined under a microscope [68].

### Blood and tissue sampling

The blood samples stood for 1 hour after collecting and were then centrifuged at 3500 r·min^-1^; then, the supernatant was kept at -20 °C until analysis. Mouse liver tissues were dissected, followed by perfusion with ice-cold phosphate buffered saline (PBS, pH 7.4) and then washing with 0.9% normal saline (1/4 w·v^-1^) to remove the blood [69]. A homogenizer was used for tissue sample homogenization in a buffer containing 10 mmol·L^-1^ Tris, phenylmethylsulphonyl fluoride (PMSF), and NaCl. The homogenates were prepared by centrifugation at 4000 rpm for 10 minutes at 4 °C, and the supernatants were stored for further detection. The tissue protein concentration in each sample was measured with the Bradford method.

### Biochemical analysis

The SOD, MDA, CAT, T-AOC, and TBARS contents in the liver tissue were measured using commercially available assay kits. The levels of AST and ALT in serum were assessed by an ELISA assay kit (RD system) according to the manufacturer’s instructions. The levels of AGEs in the serum were determined using a commercial reagent kit. All biochemical analyses were performed according to the manufacturer's protocols.

### Real-time-polymerase chain reaction (RT-PCR)

Total RNA was purified from fresh mouse liver tissue (50-100 mg) on ice with RNA fast 1000. The purity of the total RNA was checked with a spectrophotometer and the wavelength absorption ratio (260/280 nm) was between 1.7 and 2.0 in all preparations. Reverse transcription of total RNA (0.1-5 μg) to cDNA was performed using Revert Aid First Strand cDNA Synthesis Kits in 20 μL reaction volumes at 44 °C for 1 h using a Mastercycler personal PCR machine (Eppendorf AG, Hamburg, Germany). Specific primers were designed using the Beacon Designer 4.0 software and synthesized by Sunbiotech (Beijing Sunbiotech CO., Ltd, China). Real-time PCR was performed with Maxima SYBR Green/ROX qPCR Master Mix (2×) in an IQ 5.0 system. The system automatically monitors the binding of the fluorescent dye SYBR® Green to double-stranded DNA during each cycle of PCR amplification. PCR was performed under the following conditions: (1) heating at 50 °C for 2 min; (2) followed by 95 °C for 10 min and (3) 40 PCR cycles at 94 °C for 15 s, 60 °C for 30 s, and 72 °C for 30 s. The relative amount of mRNA was calculated using the comparative Ct (ΔΔCt) equation. The β-actin gene was used as a reference for normalizing the data. The derived normalized values are the mean of three runs. The mouse and human primer sequences are listed in Table 2.

**Table 2:** Real-time PCR primer sequences.

**Gene**	**Primer Sequence**	**Product size (bp)**
**NQO1**	F: 5’-ACGACAACGGTCCTTTCCAGA-3’	142
	R: 5’-CAGAAACGCAGGATGCCACT-3’	
**HO-1**	F: 5’-GCTGGTGATGGCTTCCTTGTA-3’	219
	R: 5’-ACCTCGTGGAGACGCTTTACAT-3’	
**β-actin**	F: 5’-GTGACGTTGACATCCGTAAAGA-3’ R: 5’-GTAACAGTCCGCCTAGAAGCAC-3’	287

### Western blot analysis

To determine the effects of leonurine on the Nrf2 signalling pathway, levels of several proteins in mice liver tissue homogenate were determined by Western blot. Liver tissues were lysed by 1 ml radioimmunoprecipitation assay (RIPA) lysis buffer for the extraction of total protein samples. The tissues homogenates were collected by centrifugation at 12000 rpm for 30 min at 4 °C, and the supernatant was saved and used for the concentration determination using a Bradford protein Assay kit. Then, each sample containing the same amounts of protein (40 μg) was subjected to sodium dodecyl sulphate-polyacrylamide gel electrophoresis (SDS-PAGE) and then the proteins were transferred to polyvinylidene difluoride (PVDF) membranes. The membranes were blocked in 5% fat-free milk in Tris-buffered saline with Tween 20 (TBST, 150 mM NaCl, 20 mM Tris-HCl, and 0.1% Tween 20, pH 7.5) for 1 h at room temperature. After incubating with primary antibody overnight at 4°C, the membranes were washed three times with TBST and incubated with secondary antibodies containing HO-1 (1: 200), NQO1 (1: 1000), Lamin B2 (1: 500), and β-actin (1: 500) for 2 h at room temperature. Finally, an enhanced chemiluminescence reagent (ELC) was adopted to colour the bands; pictures of the proteins were taken and processed using the image-pro-plus 6.0 software.

### Statistical analysis

The results from the study were represented as the means ± standard deviation (SD) of at least three independent experiments. The analysis was performed with the Statistical Package Social Sciences 19.0 system (SPSS Inc., Chicago, Illinois, USA), and statistical significance between experimental groups was determined by two-way analysis of variance (ANOVA) followed by *t* tests. Differences were considered statistically significant when the *P* value was less than 0.05.
